# Experimental assessment of oxygen homeostasis during acute hemodilution: the integrated role of hemoglobin concentration and blood pressure

**DOI:** 10.1186/s40635-017-0125-6

**Published:** 2017-03-01

**Authors:** Tiffanie Kei, Nikhil Mistry, Albert K. Y. Tsui, Elaine Liu, Stephen Rogers, Allan Doctor, David F. Wilson, Jean-Francois Desjardins, Kim Connelly, C. David Mazer, Gregory M. T. Hare

**Affiliations:** 1 Department of Anesthesia, St. Michael’s Hospital, 30 Bond Street, Toronto, Ontario M5B 1W8 Canada; 2 Keenan Research Centre for Biomedical Science, Li Ka Shing Knowledge Institute, St. Michael’s Hospital, 30 Bond Street, Toronto, Ontario M5B 1W8 Canada; 3 Department of Physiology, University of Toronto, Toronto, Ontario M5S 1A8 Canada; 4 Division of Pediatric Critical Care Medicine, Washington University in Saint Louis, Saint Louis, MO 63130 USA; 5 Department of Biochemistry and Biophysics, Perelman School of Medicine, University of Pennsylvania, Philadelphia, PA 19104 USA; 6 Department of Medicine, Division of Cardiology, St. Michael’s Hospital, 30 Bond Street, Toronto, Ontario M5B 1W8 Canada

**Keywords:** Hemodilution, Anemia, Mean arterial pressure, Cardiac output, Hypotension, Partial pressure of oxygen in tissue

## Abstract

**Background:**

Low hemoglobin concentration (Hb) and low mean arterial blood pressure (MAP) impact outcomes in critically ill patients. We utilized an experimental model of “normotensive” vs. “hypotensive” acute hemodilutional anemia to test whether optimal tissue perfusion is dependent on both Hb and MAP during acute blood loss and fluid resuscitation, and to assess the value of direct measurements of the partial pressure of oxygen in tissue (P_t_O_2_).

**Methods:**

Twenty-nine anesthetized rats underwent 40% isovolemic hemodilution (1:1) (or sham-hemodilution control, *n* = 4) with either hydroxyethyl starch (HES) (*n* = 14, normotensive anemia) or saline (*n* = 11, hypotensive anemia) to reach a target Hb value near 70 g/L. The partial pressure of oxygen in the brain and skeletal muscle tissue (P_t_O_2_) were measured by phosphorescence quenching of oxygen using G4 Oxyphor. Mean arterial pressure (MAP), heart rate, temperature, arterial and venous co-oximetry, blood gases, and lactate were assessed at baseline and for 60 min after hemodilution. Cardiac output (CO) was measured at baseline and immediately after hemodilution. Data were analyzed by repeated measures two-way ANOVA.

**Results:**

Following “normotensive” hemodilution with HES, Hb was reduced to 66 ± 6 g/L, CO increased (*p* < 0.05), and MAP was maintained. These conditions resulted in a reduction in brain P_t_O_2_ (22.1 ± 5.6 mmHg to 17.5 ± 4.4 mmHg, *p* < 0.05), unchanged muscle PO_2_, and an increase in venous oxygen extraction. Following “hypotensive” hemodilution with saline, Hb was reduced to 79 ± 5 g/L and both CO and MAP were decreased (*P* < 0.05). These conditions resulted in a more severe reduction in brain P_t_O_2_ (23.2 ± 8.2 to 10.7 ± 3.6 mmHg (*p* < 0.05), a reduction in muscle P_t_O_2_ (44.5 ± 11.0 to 19.9 ± 12.4 mmHg, *p* < 0.05), a further increase in venous oxygen extraction, and a threefold increase in systemic lactate levels (*p* < 0.05).

**Conclusions:**

Acute normotensive anemia (HES hemodilution) was associated with a subtle decrease in brain tissue P_t_O_2_ without clear evidence of global tissue hypoperfusion. By contrast, acute hypotensive anemia (saline hemodilution) resulted in a profound decrease in both brain and muscle tissue P_t_O_2_ and evidence of inadequate global perfusion (lactic acidosis). These data emphasize the importance of maintaining CO and MAP to ensure adequacy of vital organ oxygen delivery during acute anemia. Improved methods of assessing P_t_O_2_ may provide an earlier warning signal of vital organ hypoperfusion.

## Background

The optimal care of critically ill patients often involves management of multiple risk factors for inadequate organ perfusion, including hypotension and anemia. Assessment of the impact of hypotension and anemia in the perioperative period demonstrates that both factors are associated with serious adverse outcomes. Interoperative hypotension has been associated with increased brain [1, 2], heart [3–5], and kidney injury [3, 6, 7] and mortality [8–10]. These outcomes often depend on the magnitude and duration of hypotension; for example, a 40% drop in mean arterial pressure (MAP) from baseline for more than 30 min has been associated with myocardial injury [5]. Perioperative and acute interoperative anemia have also been associated with similar patterns of adverse events; including evidence of brain [11, 12], heart [13, 14], and kidney injury [15, 16] and mortality [14, 16–18]. Experimental models of acute anemia suggest that inadequate microcirculatory perfusion and tissue hypoxia contribute as a mechanism of vital organ injury and mortality [19–21].

Additionally, current clinical practice often favors a restrictive fluid therapy and red blood cell (RBC) transfusion approaches in surgical and critically ill patients. While many of the completed prospective randomized clinical trials favor the non-inferiority of a restrictive transfusion threshold near a hemoglobin concentration (Hb) of 70–80 g/L [22–24], more recent analysis of these data suggest that low Hb levels in the restrictive arms of these studies may be associated with increased organ injury and mortality in specific patient populations [25, 26].

We performed an experimental study to measure the partial pressure of oxygen in tissue (P_t_O_2_) in the brain and skeletal muscle, and other parameters of systemic perfusion, under conditions of acute normotensive vs. hypotensive hemodilutional anemia to assess the combined impact of acute anemia and hypotension on tissue perfusion. We hypothesize that optimal tissue perfusion depends on multiple interactive physiological parameters, including Hb and blood pressure, during acute blood loss and fluid resuscitation.

## Methods

### Overview and preparation

The Animal Care and Use Committee at St. Michael’s hospital approved all animal protocols. Twenty-nine male Sprague-Dawley rats (Jackson laboratory) with a mean weight near 500 g were anesthetized with isoflurane 2–3% in 21% oxygen for the duration of the experiment. The trachea was intubated and the lungs were mechanically ventilated using a pressure controlled ventilator, with peak inspiratory pressure between 15 and 17 cm H_2_O and a respiratory rate between 60 and 70 breaths per minute and no additional PEEP, to a target partial pressure of arterial carbon dioxide (P_a_CO_2_) near 40 mmHg (Kent Scientific Corp., Torrington, CT 06790).

The tail artery and vein were cannulated to perform the hemodilution and to monitor MAP, arterial blood gases, and Hb by co-oximetry. A jugular venous catheter was inserted in a retrograde manner toward the right atrium to provide measurements of venous blood gases. Ventilation was monitored by arterial blood gases to maintain normoxia and normocapnia. Blood pressure, ECG, and body temperature were measured using a computerized data-acquisition system (PowerLab, ADInstruments Inc., Colorado Springs CO 80906). Rectal temperature was maintained between 35 and 36 °C using a heating plate. The partial pressure of oxygen in the tissue (brain, skeletal muscle) or P_t_O_2_ was measured using oxygen-dependent quenching of phosphorescence as previously described [27] utilizing a novel microsensor G4 Oxyphor technology (PMOD1000 instrument, Oxygen Enterprises, Ltd., Philadelphia, PA 19104–1808).

### Hemodilution protocol

After performing all of the procedures, baseline measurements were collected for 10 min. Rats then underwent either “normotensive” hemodilution with 1:1 volume exchange of 40% estimated total blood volume (30 ml/kg) with either 6% hydroxyethyl starch (HES) 130/0.4 in 0.9% sodium chloride (Voluven, *n* = 14, Fresenius Kabi Canada, Mississauga ON L4W 4Y3), or “hypotensive” hemodilution with 0.9% sodium chloride (saline, *n* = 11). During hemodilution, arterial blood was exchanged with HES or saline at a fixed rate over 10 min, using the push-pull infusion pump (PHD2000, Harvard Apparatus Canada, St. Laurent, Quebec, H4S 1R9). Following hemodilution, physiologic parameters were continuously acquired during a 60-min recovery period. Brain and hind limb skeletal muscle tissue P_t_O_2_ were recorded continuously. Arterial and venous blood gases and co-oximetry measurements were collected at baseline, immediately following hemodilution, and at 30 and 60 min following completion of hemodilution using a heparinized syringe. Samples were analyzed using a blood gas analyzer and co-oximeter (ABL 800, Radiometer Canada, London ON, N5V 4T7).

An additional four rats were placed in a sham control group and did not undergo hemodilution, to serve as a time-based negative control group.

### Cardiac output measurements

These experiments included four rats per hemodilution group, which underwent cardiac output (CO) measurements before and after hemodilution. Transthoracic echocardiography was performed in hemodiluted rats in the supine position at baseline and within 15 to 30 min following hemodilution utilizing a high-frequency ultrasound system (Vevo 2100, MS-250 transducer, Visualsonics, Toronto, ON). Two dimensional long-axis images of the left ventricle in parasternal long- and short-axis views with M-mode measurements at mid-papillary muscle level and linear dimensions were analyzed offline (Vevo 2100 software v. 1.3) using the standard leading edge to leading edge technique. CO was calculated as SV × HR, where SV and HR are stroke volume and heart rate, respectively. SV and ejection fraction (EF) were measured using the Teicholz cubed formula, LV volume = 7 × LVID^3^/(2.4 + LVID), followed by the difference between ESV and EDV, where LVID, ESV, and EDV are left ventricular internal diameter, end systolic volume, and end diastolic volume, respectively. Fractional shortening (FS%) was calculated as (LVIDd − LVIDs)/LVIDd × 100, where LVIDd and LVIDs are left ventricular end diastolic and end systolic internal diameters, respectively. Three consecutive cardiac cycles were averaged for all analyses.

### Statistical analysis

All data are presented as mean ± SD. Data were analyzed by repeated measures two-way ANOVA and post hoc Tukey test, where appropriate according to Sigmaplot software (SigmaStat 11.0).

## Results

Baseline values were comparable for all parameters measured between the three experimental and control groups. No differences in heart rate (299.0 ± 29.5, 309.7 ± 51.8, 290.5 ± 3.2 bpm) or body temperature (36.1 ± 0.5, 36.0 ± 0.4, 35.9 ± 0.3 °C) were observed at baseline or at any time point for the HES, saline, and sham control groups, respectively. Arterial and venous blood gases and Hb concentrations are reported in Tables 1 and 2.

**Table 1:** Arterial and venous blood gases following hemodilution

		Arterial blood samples				Venous blood samples			
		Baseline	Post-hemodilution	30 min	60 min	Baseline	Post-hemodilution	30 min	60 min
Hb (g/L)	Control	123 ± 10	121 ± 9	110 ± 2	101 ± 8*	126 ± 5	120 ± 5	113 ± 6*	106 ± 8*
	HES	131 ± 9	66 ± 6*$	63 ± 6*$	60 ± 8*$	130 ± 8	58 ± 8*$	61 ± 6*$	60 ± 7*$
	Saline	130 ± 11	79 ± 5*$#	75 ± 11*$	75 ± 14*$	125 ± 12	75 ± 7*$#	74 ± 11*$#	79 ± 12*$#
sO_2_ (%)	Control	90.9 ± 0.8	89.0 ± 2.5	88.5 ± 2.6	83.5 ± 8.6	65.6 ± 8.4	60.5 ± 8.0	58.5 ± 4.3	51.8 ± 6.5*
	HES	87.4 ± 3.8	85.9 ± 4.8	84.2 ± 6.6	87.5 ± 8.9	62.7 ± 3.7	55.3 ± 4.3	49.2 ± 5*	43 ± 4.6*
	Saline	88 ± 2.9	90.8 ± 3.4	92.4 ± 3.1	89.0 ± 8.0	64.1 ± 5.7	37.7 ± 9.9*$#	31.7 ± 11.9*$#	18.3 ± 7.3*$#

*p < 0.001 vs. baseline, $*p* < 0.001 vs. control, #*p* < 0.001 vs. HES and control

**Table 2:** Arterial and venous blood gases following starch (HES) and saline hemodilution

		Arterial blood samples				Venous blood samples			
		Baseline	Post-hemodilution	30 min	60 min	Baseline	Post-hemodilution	30 min	60 min
pH	Control	7.35 ± 0.03	7.32 ± 0.02	7.33 ± 0.01	7.31 ± 0.02	7.33 ± 0.04	7.30 ± 0.03	7.30 ± 0.02	7.28 ± 0.02
	HES	7.37 ± 0.02	7.34 ± 0.02	7.36 ± 0.03	7.37 ± 0.04	7.35 ± 0.02	7.33 ± 0.02	7.33 ± 0.02	7.33 ± 0.04
	Saline	7.37 ± 0.04	7.34 ± 0.04	7.31 ± 0.07	7.24 ± 0.18*	7.35 ± 0.03	7.28 ± 0.05	7.26 ± 0.1*	7.08 ± 0.15*$#
pCO_2_ (mmHg)	Control	39.3 ± 4.5	40.3 ± 2.6	38.7 ± 2.5	41.5 ± 5.1	44.2 ± 7.7	47.8 ± 5.1	45.6 ± 2.6	46.9 ± 5.7
	HES	39.6 ± 5.2	41.7 ± 3.4	40.5 ± 4.7	37.0 ± 6.2	44.3 ± 3.5	44.3 ± 4.3	42.7 ± 4.7	42.5 ± 5.3
	Saline	37.9 ± 3.8	36.6 ± 4.1	28.3 ± 4.1*$#	25.3 ± 10*$#	41.7 ± 4.1	44.8 ± 4.8	39.6 ± 8.7	46.6 ± 5.8
pO_2_ (mmHg)	Control	88.1 ± 6.1	87.2 ± 6.9	84.3 ± 8.4	82.3 ± 22.4	50 ± 8.3	49 ± 5.3	46.8 ± 4.0	41.9 ± 3.9*
	HES	77.3 ± 8.3	76 ± 10.6	71.9 ± 13.6	82 ± 18.4	47.4 ± 2.6	42.3 ± 2.8*	38.6 ± 4.3*$	34.7 ± 2.5*
	Saline	79.8 ± 8.6	87.7 ± 15.1	103.2 ± 10.4*	88.4 ± 18.5	47.9 ± 4.2	33.1 ± 5.9*$#	32.5 ± 7.5*$	23.6 ± 5.1*$#
HCO_3_^-^ (mmol/L)	Control	20.9 ± 2.1	20.3 ± 1.3	19.7 ± 0.7	20.3 ± 1.7	22.5 ± 2.9	22.6 ± 1.9	21.6 ± 1.4	21.5 ± 2.3
	HES	22.5 ± 2.0	22.3 ± 1.8	22.1 ± 1.8	20.8 ± 1.6	23.8 ± 1.3	22.5 ± 1.9	21.7 ± 1.7*	21.6 ± 1.4*
	Saline	21.5 ± 0.8	19.2 ± 0.8	13.9 ± 1.9*$#	10.8 ± 5.4*$#	22.5 ± 1.6	20.4 ± 1.3*	16.8 ± 1.8*$#	13.7 ± 3.6*$#
Lactate (mmol/L)	Control	2.4 ± 0.6	2.7 ± 0.6	2.6 ± 0.73	2.7 ± 0.7	2.5 ± 0.6	3.0 ± 0.3	3.0 ± 0.4	2.9 ± 1.0
	HES	2.5 ± 0.5	2.5 ± 0.7	2.8 ± 0.9	3.2 ± 0.9	2.5 ± 0.5	2.4 ± 0.6	2.4 ± 0.6	2.8 ± 0.7
	Saline	3.5 ± 1.3	3.3 ± 1.2	6.5 ± 2.2*$#	10.9 ± 6.2*$#	3.1 ± 1.0	3.8 ± 1.0	6.4 ± 1.7*$#	10.6 ± 5.1*$#

*p < 0.001 vs. baseline, $*p* < 0.001 vs. control, #*p* < 0.001 vs. HES and control

### Arterial and venous co-oximetry and blood gas analysis

In the normotensive hemodilution (HES) group, mean Hb values decreased from a baseline of 131 ± 9 to 66 ± 6 g/L immediately following hemodilution (*p* < 0.001). In the hypotensive hemodilution saline group, Hb decreased from 130 ± 11 to 79 ± 5 g/L (Table 1; *p* < 0.001). The Hb concentration in the saline hemodilution group was initially higher than in the HES hemodilution group (Table 1). In the sham control rats, Hb did not decrease over time.

**Fig. 1:**
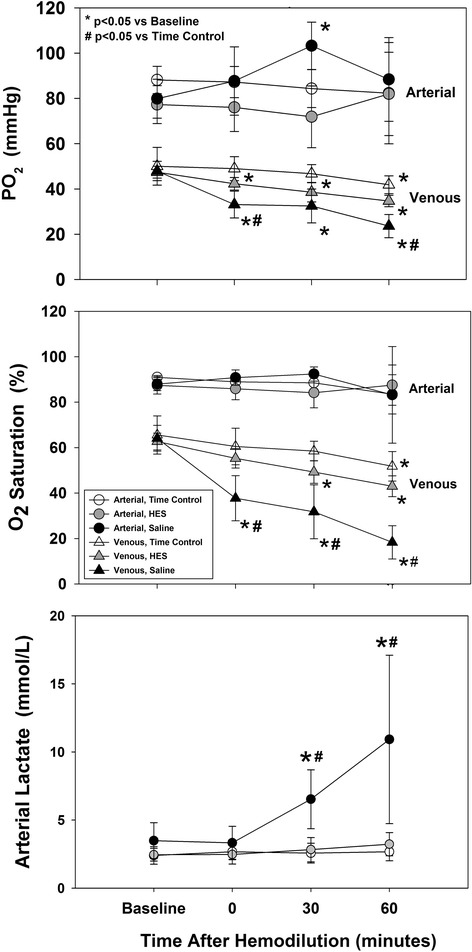
*Upper panel* Arterial PO_2_ remained stable except for a transient increase at 30 min (associated with hyperventilation) in the saline hemodilution group. A significant reduction in venous PO_2_ was observed in the saline hemodilution group relative to the controls (*p < 0.001 vs. baseline, #*p* < 0.05 vs. control). *Middle panel* Arterial oxygen saturation remained stable in all groups. A significant reduction in venous PO_2_ was observed in all groups relative to baseline. The venous oxygen saturation in the saline hemodilution group was reduced relative to the controls and HES group (*p < 0.001 vs. baseline, #*p* < 0.05 vs. control and HES). *Lower panel* A significant rise in arterial lactate was only observed in the saline hemodilution group (*p < 0.001 vs. baseline, #*p* < 0.05 vs. control and HES)

Arterial oxygen tension (P_a_O_2_) and Hb saturation remained stable and did not decrease throughout the experimental protocol in any group (Tables 1 and 2, Fig. 1). Venous oxygen tension (P_v_O_2_) and saturation decreased from baseline after 60 min in all groups. There was no difference between P_v_O_2_ between the sham controls and the HES hemodilution group. By contrast, there was a more profound decrease in P_v_O_2_ and saturation in the saline hemodiluted group (Tables 1 and 2, Fig. 1, *p* < 0.05). Following saline hemodilution, the P_a_O2 was increased transiently at 30 min following hemodilution. This change was associated with a reduction in P_a_CO_2_ (Table 2; *p* < 0.001), which is consistent with a respiratory compensation to metabolic acidosis. Animals in this group developed a lactic acidosis, as indicated by a significant increase in lactate, a reduction in pH, and HCO_3_^−^ (Table 2, Fig. 1, *p* < 0.001 for all). A maximal rise in arterial lactate was achieved by 60 min following hemodilution (3.5 ± 1.3 to 10.9 ± 6.2 mmol/L, *p* < 0.05).

### Mean arterial blood pressure measurements

**Fig. 2:**
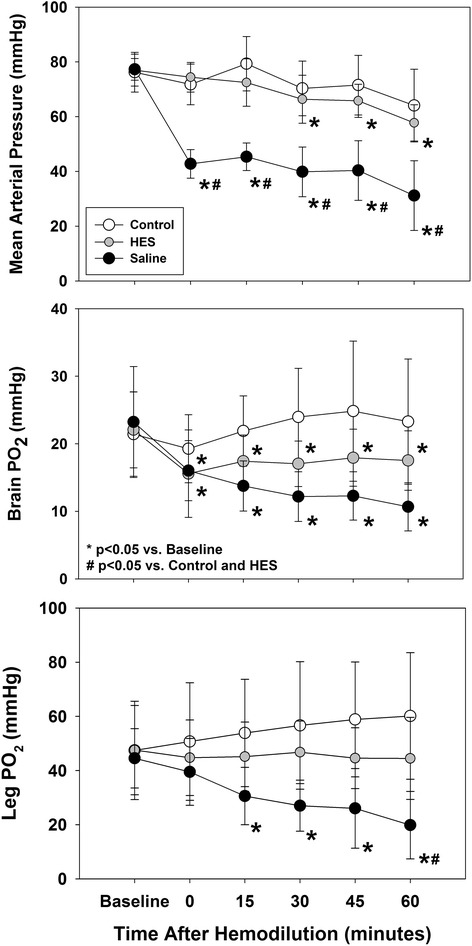
*Upper panel* Mean arterial pressure remained stable in controls and after hemodilution with hydroxyethyl starch (HES) but decreased significantly, relative to controls and HES hemodilution groups, following hemodilution with saline (*p < 0.001 vs. baseline, #*p* < 0.05 vs. control and HES). *Middle pane*l Brain PO_2_ decreased from baseline following hemodilution with both HES and saline (*p < 0.001 vs. baseline). *Lower panel* Skeletal muscle PO_2_ remained stable in control and HES hemodilution groups but was reduced relative to baseline and controls after saline hemodilution (*p < 0.001 vs. baseline, #*p* < 0.05 vs. control and HES)

There was a significant treatment, time, and interaction effect (two-way ANOVA; *p* < 0.001 for all). MAP was maintained for the duration of the 60-min recovery period in both the normotensive hemodilution and control group (Fig. 2). By contrast, there was an immediate decrease in MAP following hemodilution in the saline hemodilution group (77.3 ± 4.0 vs. 42.8 ± 5.2 mmHg, *p* < 0.05), which persisted to the 60-min recovery period (31.1 ± 12.8 mmHg). MAP was significantly lower in the saline hemodilution group, relative to both controls and starch hemodilution groups at all post-hemodilution time points (Fig. 2, *p* < 0.001).

### Partial pressure of oxygen in brain and muscle tissue (P_t_O_2_)

There was a significant time and interaction effect for brain P_t_O_2_ when comparing all three experimental groups (two-way ANOVA; *p* < 0.001 for both). Brain P_t_O_2_ remained stable in the time-based control sham hemodiluted group. Following normotensive hemodilution with HES, brain P_t_O was reduced from a baseline value of 22.1 ± 5.6 mmHg to a value of 15.6 ± 6.5 mmHg immediately following hemodilution. The brain P_t_O_2_ remained decreased for 60 min reaching a value of 17.5 ± 4.4 mmHg (Fig. 2, *p* < 0.05). Rats undergoing hypotensive hemodilution with saline experienced a greater decrease in brain P_t_O_2_ which decreased from baseline (23.2 ± 8.2 mmHg) to values as low as 16.0 ± 4.5 mmHg immediately following hemodilution. The brain P_t_O_2_ reached a nadir value of 10.7 ± 3.6 mmHg after 60 min (Fig. 2, *p* < 0.05).

There was no time or interaction effect observed for hind limb skeletal muscle PO_2_ (*p* = 0.051 and 0.082, respectively). However, there was a significant time-treatment interaction (two-way ANOVA; *p* < 0.001). No change in muscle P_t_O_2_ was observed following sham procedure or HES hemodilution (Fig. 2). By contrast, muscle PO_2_ decreased significantly from baseline values of 44.5 ± 11.0 mmHg to 30.6 ± 10.6 mmHg immediately following hemodilution with saline (Fig. 2, *p* < 0.05). In this group, muscle P_t_O_2_ reached a nadir at 60 min (19.9 ± 12.4 mmHg, *p* < 0.05).

### Cardiac output measurements

CO data from normotensive and hypotensive hemodilution groups are presented in Table 3. These experiments demonstrate that CO is increased in the normotensive HES hemodilution group, predominantly due to an increase in diastolic volume and stroke volume. These changes were not observed in the hypotensive saline hemodilution group which demonstrated a decrease in CO, SV, and diastolic volume (Table 3).

**Table 3:** ECHO measures following hemodilution

		Baseline	Post-hemodilution
MAP (mmHg)	HES	82.2 ± 19.6	74.3 ± 19.7
	Saline	103.6 ± 12.6	60.0 ± 5*
Hb (g/L)	HES	144 ± 6	77 ± 7*
	Saline	136 ± 3	88 ± 10*#
CO (mL/min)	HES	57.7 ± 8.7	116.8 ± 15.6*
	Saline	72.8 ± 22.4	41.1 ± 14.0*#
HR (bpm)	HES	323.6 ± 46.1	382.0 ± 29.0
	Saline	362.9 ± 22.7	333.0 ± 23.0
SV (μL)	HES	179.9 ± 26.9	305.7 ± 32.2*
	Saline	199.1 ± 55.5	122.1 ± 33.8*#
Diastole volume (μL)	HES	217.5 ± 45.1	331.8 ± 30.8*
	Saline	263.5 ± 70.2	149.8 ± 33.1*#
Systole volume (μL)	HES	37.6 ± 21.9	26.1 ± 12.1
	Saline	64.4 ± 16.6	27.7 ± 11.2
EF (%)	HES	83.5 ± 6.6	92.1 ± 3.6
	Saline	75.3 ± 2.8	81.1 ± 8.7
FS (%)	HES	54.2 ± 7.4	66.8 ± 6.6
	Saline	45.4 ± 2.8	51.1 ± 8.7

*p < 0.01 vs. baseline, #*p* < 0.05 vs HES; two-way ANOVA repeated measures

## Discussion

We demonstrated a significant interaction between low Hb concentration and low MAP with respect to limiting tissue oxygen delivery in brain and muscle, in a model of normotensive vs. hypotensive hemodilution. During normotensive hemodilution with HES, blood pressure was maintained, CO increased, and systemic perfusion was generally preserved, as indicated by the absence of lactic acidosis and sustained muscle P_t_O_2_. However, under these conditions, brain tissue P_t_O_2_ was significantly reduced, suggesting that tissue oxygen delivery did not meet the higher metabolic requirements for oxygen in the brain. These data are consistent with our previous studies, which demonstrate that acute normotensive anemia is associated with brain tissue hypoxia and activation of hypoxic cellular signaling pathways, including hypoxia inducible factor (HIF) [20]. This data may help to explain why acute hemodilution and anemia are associated with increased stroke incidence in patients undergoing cardiac and non-cardiac surgery [11, 12]. In addition, acute anemic conditions which produce mild brain tissue hypoxia have been associated with evidence of more severe renal tissue hypoxia [21, 28]; providing a plausible explanation for both stroke acute kidney injury (AKI) in patient exposed to hemodilutional anemia [12, 16]. The combined effect of inadequate perfusion to vital organs during acute anemia may contribute to the observed association of increased mortality in anemic perioperative patients [16, 17].

By contrast, hemodilution with saline resulted in hypotension, a reduction in CO, a further reduction in brain P_t_O_2_, a newly observed reduction in muscle P_t_O_2_, and a severe increase in lactate suggestive of profound systemic ischemia. These data demonstrate the additive impact of combined low Hb in the face of a low CO and low MAP on tissue oxygen delivery in an experimental model. The mechanism likely involves inadequate fluid resuscitation with reduced intravascular volume and an inadequate cardiovascular response to anemia as indicated by the CO and diastolic volume analysis. From prior studies, we understand that during acute anemia, oxygen homeostasis is maintained by an active increase in CO to ensure perfusion of vital organs including the brain [19, 20]. Inhibition of the CO response results in accentuation of tissue hypoxia [19]. We observed a similar effect in the saline hemodilution group where an inadequate CO response to anemia resulted in profound tissue hypoxia further demonstrating the need to preserve adequate intravascular fluid volume during anemia.

### Can measurement of tissue PO_2_ inform clinical practice to improve outcomes?

Clinical monitoring of the partial pressure of oxygen in brain tissue has been performed directly using invasive tissue probes in patients exposed to neurotrauma; however, the invasive nature of these probes severely limits their use clinically. Indirect assessment of brain P_t_O_2_ has been achieved utilizing near infrared spectroscopy (NIRS). NIRS measures changes in arterial and venous oxy- and deoxyhemoglobin levels which indirectly reflect levels of microvascular oxygenation. NIRS is capable of detecting cerebral microvascular oxygen desaturation in patients undergoing heart surgery [6, 29–31]. Treatment algorithms have been defined to respond to, and correct, episodes of cerebral desaturation [29, 31]. While these maneuvers are able to correct the observed cerebral desaturations, only one study has demonstrated improvement in patient outcome [31].

Other novel light-based methods for directly measuring cellular energetics and tissue PO_2_ are being developed. Spectroscopic approaches, including broadband spectroscopy, allowed for measurement of the oxidative state of cellular cytochromes which reflect cellular energetics [32, 33]. Measurement of cutaneous mitochondrial PO_2_, utilizing the oxygen-dependent delayed fluorescence of protopor-phyrin IX in the skin [34, 35], has been used to assess the mitochondrial PO_2_ response to acute hemodilution in an experimental pig model [35]. This approach was able to detect tissue hypoxia at an earlier stage of hemodilution relative to more traditional measurements including changes in MAP, serum lactate, VO_2_, and NIRS [35]. Future studies will be required to assess the impact of these technologies on patient outcomes.

In clinical studies, both hypotension [1–10] and anemia [11–18] have been independently associated with increase acute renal injury [3, 6, 7, 15, 16], myocardial infarction [3–5, 13, 14], stroke [1, 2, 11, 12], and mortality [8–10, 14, 16–18]. However, few of these retrospective analyses have formally assessed the combined impact of low Hb and low MAP on patient outcome. A 2012 post hoc analysis by Haase et al. demonstrated a 3.36 (1.34–8.41)-fold increase in cardiac surgery risk of AKI associated with a combination of anemia and hypotension during CPB relative to anemia alone [36]. However, a retrospective study by Sickeler et al. in 2014, which intended to replicate Haase’s findings, involved a much larger cohort of 3963 patients and did not find any association between the co-occurrence of hypotension and anemia in cardiac surgery-related AKI risk [37]. Further clinical research is needed to fully assess the potential interaction between anemia and hypotension and their combined impact on patient outcome.

### Optimal choice of resuscitation fluid in critical care

Data to support the choice of optimal fluid for resuscitation (blood, colloid, crystalloid, blood substitute) in specific critical care settings is lacking [38]. While a complete review is beyond the scope of this manuscript, our data support some interesting observations about fluid restriction and the potential value of monitoring tissue P_t_O_2_ to assess fluid replacement and transfusion strategies. Recent assessment of clinical practice favors goal-directed and/or restrictive fluid management strategies [39]. In addition, review of management strategies in critical care demonstrate a preference toward crystalloid as the first choice for fluid therapy [40] and an overall increase in vasopressor use [41]. This combination of treatment strategies may lead to an increased risk of patients with reduced intravascular volume and support the priority of increased utilization of monitors which assess the adequacy of microvascular perfusion of vital organs [42].

Data from prospective randomized trials assessing restrictive vs. liberal RBC transfusion thresholds have largely favored a restrictive approach [43]. More recent analysis suggest that some patient populations may be harmed by this restrictive approach including patients undergoing cardiac surgery [44, 45] or those experiencing acute myocardial ischemia [25]. Data from the TITRe2 trial demonstrated that patients randomized to a restrictive transfusion threshold (Hb <7.5 g/dL) experienced a higher mortality [(4.2 vs. 2.6%; HR 1.64 (1.00 to 2.679)], relative to patients randomized to a liberal threshold [44]. Early data from the TRICS trial in cardiac surgery also demonstrated a trend to increased adverse events including stroke (3 vs. 0) and death (4 vs. 1) in the restrictive study arm [46]. Utilization of methods to directly assess tissue oxygen delivery may help to define appropriate patient-specific fluid therapy and RBC transfusion thresholds in different patient populations. Finally, direct measurement of tissue oxygen delivery may promote the development of novel blood substitutes, including hemoglobin-based oxygen carriers (HBOCs). In experimental models, HBOCs have been demonstrated to maintain oxygen delivery to tissue during severe degrees of volume exchange [47]. However, due to concerns about toxicity and adverse clinical outcomes associated with HBOC use [48], future development of HBOCs will require measures of both efficacy (P_t_O_2_) and safety.

There are several limitations to the current study. We did not provide a whole blood exchange control, as these controls had been performed previously without any effect on tissue oxygen measurements relative to time-based controls [49]. We did not directly assess changes in intravascular volume. Further, although crystalloids and colloids are used clinically, they are typically not used comparably in a direct 1:1 blood volume exchange. Thus, our saline hemodilution group likely resulted in additional hemodynamic stress, including reduced intravascular volume that is not reflective of clinical care.

## Conclusions

In this study, we observed that hypotensive anemia (1:1 saline/blood fluid exchange) resulted in global ischemia and severe tissue hypoxia. By contrast, normotensive anemia (1:1 HES/blood fluid exchange) preserved global organ perfusion but was unable to prevent a subtle reduction in brain tissue P_t_O_2_. These data support the ongoing assessment of clinically applicable technologies to assess and measure tissue P_t_O_2_, in order to develop strategies maintaining tissue oxygen delivery and limiting adverse events associated with tissue hypoxia in patients with critical illness.

## Abbreviations

Hb: Hemoglobin
HES: Hydroxyethyl starch
MAP: Mean arterial pressure
PCO_2_: Partial pressure of carbon dioxide
PO_2_: Partial pressure of oxygen
